# Molecular characterization of metabolic subtypes of gastric cancer based on metabolism-related lncRNA

**DOI:** 10.1038/s41598-021-00410-7

**Published:** 2021-11-02

**Authors:** Lingdi Li, Jianfei Ma

**Affiliations:** 1 Internal Medicine-Oncology, Hangzhou Cancer Hospital, Hangzhou, China; 2 Key Laboratory of Image Information Processing and Intelligent Control, School of Artificial Intelligence and Automation, Huazhong University of Science and Technology, Luoyu Road 1037, Wuhan, 430074 China

**Keywords:** Cancer epidemiology, Cancer metabolism, Cancer therapy, Gastrointestinal cancer

## Abstract

Increasing evidence has demonstrated that lncRNAs are critical regulators in diverse biological processes, but the function of lncRNA in metabolic regulation remains largely unexplored. In this study, we evaluated the association between lncRNA and metabolic pathways and identified metabolism-related lncRNAs. Gastric cancer can be mainly subdivided into 2 clusters based on these metabolism-related lncRNA regulators. Comparative analysis shows that these subtypes are found to be highly consistent with previously identified subtypes based on other omics data. Functional enrichment analysis shows that they are enriched in distinct biological processes. Mutation analysis shows that ABCA13 is a protective factor in subtype C1 but a risk factor in C2. Analysis of chemotherapeutic and immunotherapeutic sensitivity shows that these subtypes tend to display distinct sensitivity to the same chemical drugs. In conclusion, these findings demonstrated the significance of lncRNA in metabolic regulation. These metabolism-related lncRNA regulators can improve our understanding of the underlying mechanism of lncRNAs and advance the research of immunotherapies in the clinical management of gastric cancer.

## Introduction

The human genome contains about 20,000 protein-coding genes, whereas most of the genome is transcribed into non-coding RNA^1,2^. Long non-coding RNAs (lncRNA), defined as transcripts over 200 nucleotides, do not have protein-coding potential^3^. They participate in the regulation of gene expression at different levels^4^. In recent years, a growing number of researches have focused on the identification of the function of lncRNA. LncRNA is found to be significantly associated with diverse biological processes, such as cell differentiation, proliferation, and apoptosis^5–7^. Moreover, human immunity is reported to be regulated by lncRNA^8^. But most of the lncRNAs remain largely unexplored and the function of these lncRNAs is still unknown. Therefore, it’s of great significance to identify the function of lncRNA.

Growing researches have demonstrated the association between lncRNA and human cancer. LncRNA MEG3 is found to inhibit proliferation and metastasis of gastric cancer via p53 signaling pathway^9^. LncRNA MT1JP is reported to function as a ceRNA in regulating FBXW7 through competitively binding to miR-92a-3p in gastric cancer^10^. Additionally, lncRNA is found to be significantly associated with immune pathways of primary human cancer^11^. But most of these analyses concentrated on a single lncRNA, functional characterization of lncRNA is still inadequate. It’s necessary to investigate the lncRNA that has not previously been studied.

Currently, an increasing number of studies demonstrated the significance of metabolism in human cancer. Initiation and progression of colorectal cancer are found to be regulated by metabolic pathways^12^. Cancer metabolism research revealed that metabolic profiles of individual tumors are highly heterogenous^13^ and metabolic expression subtypes of human cancers are significantly associated with patient survival^14^. But all of these studies concentrated on protein-coding genes. The regulation of lncRNA on metabolic pathways is unknown. Thus, it’s of great interest to study the underlying interactions between lncRNA and metabolic pathways. Deeply understanding the function of these metabolism-related lncRNAs can provide insights into the molecular mechanism of lncRNA.

In this work, we aim to identify the interactions between metabolism and lncRNA and analyze the clinical relevance of these metabolism-related lncRNAs. After evaluating the association between lncRNA and metabolic pathways, we identified 1539 metabolism-related lncRNAs. Gastric cancer can be mainly classified into 2 subtypes based on these lncRNAs. Functional enrichment analysis shows that they are enriched in distinct biological processes. Genomic characterization shows that ABCA13 mutation is a protective factor in C1 but a risk factor in C2. Analysis of chemotherapeutic and immunotherapeutic sensitivity shows that different subtypes tend to display distinct sensitivity to the same chemical drugs, suggesting the clinical significance of these metabolism-related lncRNAs.

## Result

### Characterization of metabolism-related lncRNA

**Figure 1:**
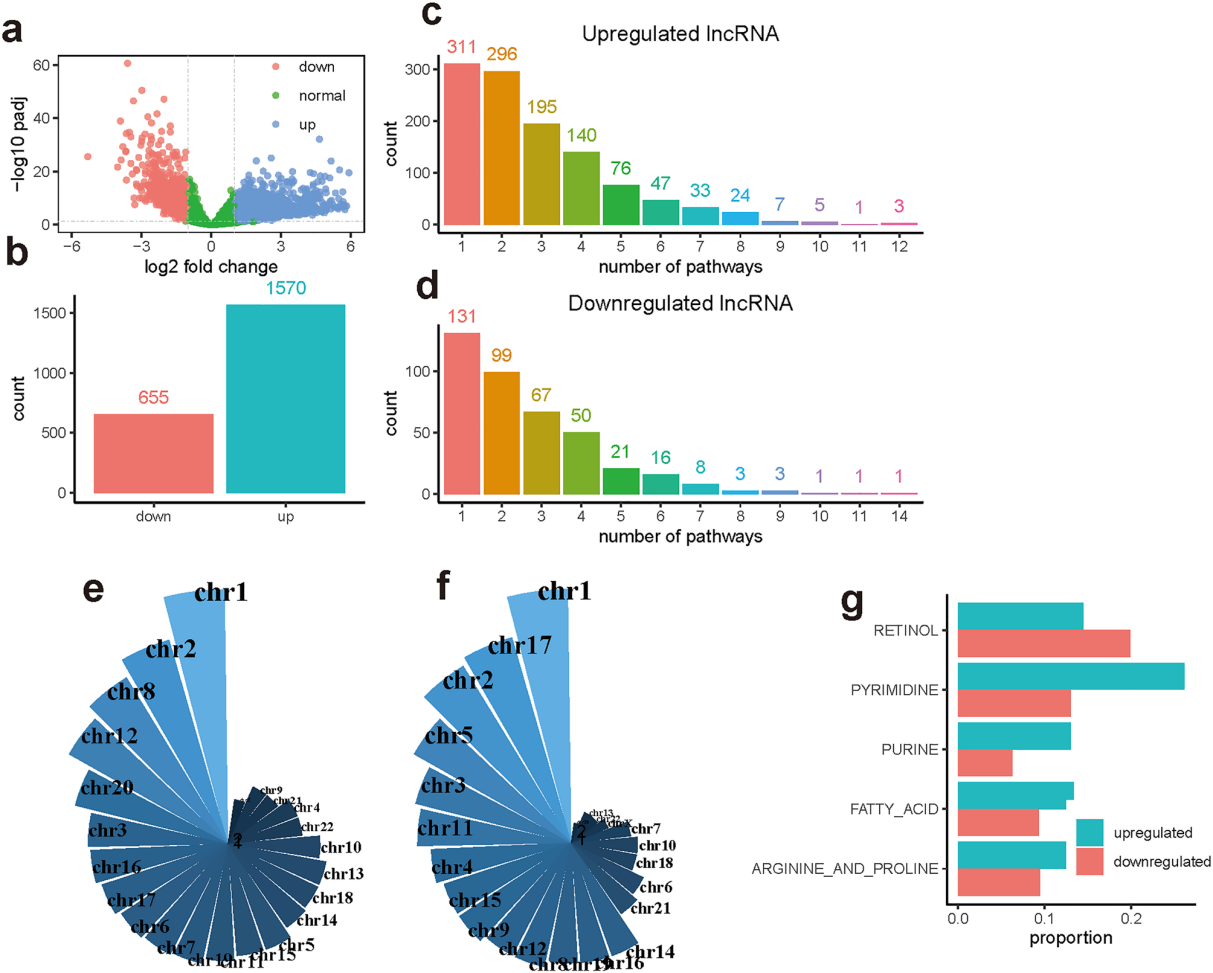
Characterization of metabolism-related lncRNA in gastric cancer. (**a**) Identification of differentially expressed lncRNAs between gastric cancer and the adjacent normal samples. Red and blue nodes represent the downregulated and upregulated lncRNAs respectively. (**b**) Barplot shows the number of upregulated and downregulated lncRNA respectively. (**c**, **d**) Summary of lncRNA based on the number of associated metabolism pathways. Chromosomal distribution of these upregulated (**e**) and downregulated (**f**) lncRNAs that are significantly associated with the metabolism pathway. (**g**) Top 5 metabolic pathways that are frequently regulated by lncRNA.

We performed differential expression analysis between gastric cancer and the adjacent normal samples and identified 1570 upregulated lncRNAs and 655 downregulated lncRNAs (Fig. 1a, b). Association analysis was then used to evaluate the interactions between these dysregulated lncRNAs and metabolic pathways which is implemented using R package ImmLnc^11^. Among these upregulated lncRNAs, 1138 (72.5%) lncRNAs are associated with at least one metabolic pathway and identified as metabolism-related lncRNAs (Fig. 1c). For these downregulated lncRNAs, 401 (61.2%) lncRNAs are identified as metabolism-related lncRNAs (Fig. 1d). These significant lncRNA-metabolic pathway pairs are presented in Supplementary Table 1. In addition, we found that the number of lncRNAs is negatively associated with the number of their associated metabolic pathways (Fig. 1c, d). Most of the lncRNAs are associated with a few metabolic pathways. It suggests that only a small proportion of lncRNAs play significant roles in the regulation of metabolic pathways.

To explore the significant value of these lncRNAs in gastric cancer, we investigated these metabolism-related lncRNAs on the published literatures and found that some of them have been reported to be associated with tumorigenesis. For example, lncRNA H19, which is identified as the most variable gene in our analysis, is found to be associated with various cancer types. It is also found to regulate various metabolic pathways including energy metabolism and glucose metabolism^15,16^. AFAP-AS1 is reported to regulate trastuzumab resistance of breast cancer^17^. MAP3K20-AS1 is found to inhibit gastric cancer growth through epigenetically regulating miR-375^18^. Other lncRNA including ELFN1-AS1, CASC9, SLCO4A1-AS1, DSCR8 and so on are all found to be studied previously and associated with at least one cancer type, which demonstrates that these lncRNAs can indeed capture the oncogenic features of gastric cancer.

Regional analysis of these metabolism-related lncRNAs shows that both upregulated and downregulated lncRNAs are preferentially located on chromosomes 1 and 2 (Fig. 1e, f). Comparative analysis shows that upregulated lncRNAs are preferentially located on chromosomes 8 and 12, whereas downregulated lncRNAs are preferentially located on chromosomes 17 and 5. Furthermore, we ranked the metabolic pathways by the number of associated lncRNAs and investigated the pathways that are frequently regulated by lncRNA. Retinol metabolism, pyrimidine metabolism, purine metabolism, fatty acid metabolism, and arginine and proline metabolism are identified as the top 5 metabolic pathways (Fig. 1g). In conclusion, these results demonstrate that lncRNA is significant in the regulation of metabolic activities.

### Molecular classification of gastric cancer based on metabolism-related lncRNA

**Figure 2:**
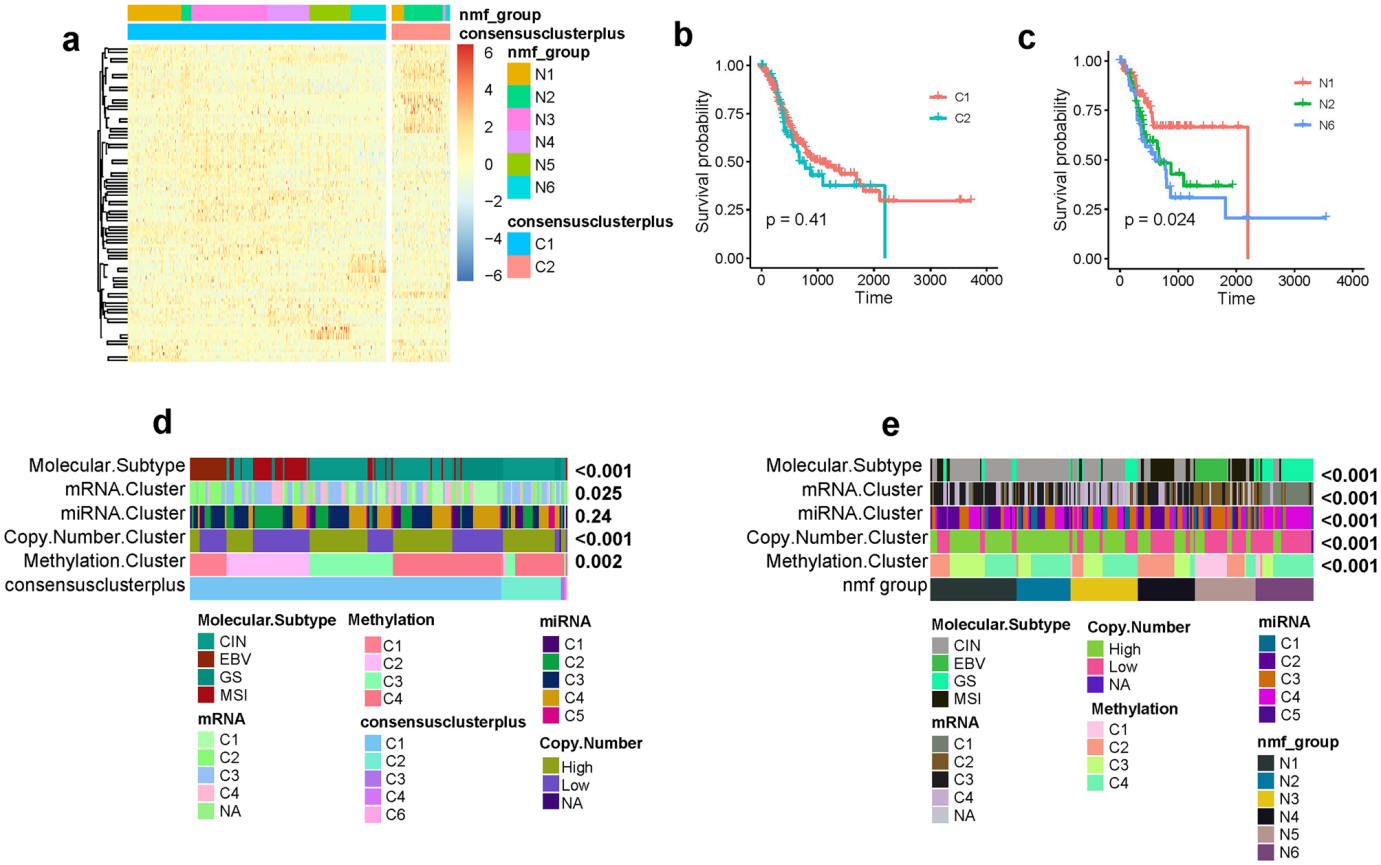
Molecular classification of gastric cancer based on these metabolism-related lncRNAs. (**a**) Heatmap shows the molecular subtypes of gastric cancer using two independent methods consensusclusterplus and non-negative matrix factorization based on the top 100 variable lncRNAs. (**b**, **c**) Survival analysis of gastric cancer between different subtypes. The difference of survival curve is evaluated using log-rank test. (**d**, **e**) Comparative analysis of different gastric cancer subtypes. The association between our metabolism subtypes and the previously identified subtypes is evaluated using chisquare test.

These metabolism-related lncRNAs are used to classify gastric cancer into different subtypes. R package ConsensusClusterPlus^19^ is used for the molecular classification of gastric cancer. To determine the optimal number of clusters of gastric cancer, we start a cluster survey from 2 to 8. After comprehensively evaluating the cumulative distribution function curve and consensus matrix, k = 6 is identified as the optimal number of clusters. Due to the small number of samples in cluster C3–C6, we removed these clusters and yielded 2 clusters C1 and C2. To evaluate the reliability of molecular subtypes of gastric cancer, an independent approach called non-negative matrix factorization (NMF)^20^ yielded 6 clusters of gastric cancer (N1–N6). Comparative analysis between these subtypes shows that the molecular subtypes derived from these two approaches achieve high consistency. Cluster N2 is highly enriched in cluster C2 (Fig. 2a). Survival analysis between different subtypes shows that subtype N1 has a favorable prognosis compared with subtype N2 and N6 (Fig. 2c). But no survival difference is observed between ConsensusClusterPlus subtypes (Fig. 2b).

To check whether there is a similarity between our lncRNA subtypes and the subtypes derived from other omics data, we investigated these lncRNA based metabolic subtypes with previously identified subtypes based on mRNA expression, miRNA expression, methylation, and copy number variation and found that they achieve high consistency^21^. Some subtypes derived from different omics data overlapped highly with each other (Fig. 2d, e). For example, ConsensuscluaterPlus-based subtype C1 is highly overlapped with EBV and MSI subtypes; C1 is found to be overlapped with CNV low group, methylation C1, C2, and C3 groups, mRNA C1, C2, and C4 groups (Supplementary Fig. 1). Furthermore, the association of NMF-based subtypes is also explored. Subtype N1 is highly overlapped with CIN subtype, mRNA subtype C3, and CNV high group; N2 is preferentially overlapped with CIN subtype, mRNA subtype C3, and CNV high group. N3 is found more likely to be overlapped with CIN and GS subtypes, mRNA subtype C4. N6 is found to be overlapped with GS subtype, mRNA C1 group, miRNA C4 group, methylation C4 group and CNV low group. The overlapped interactions between lncRNA based metabolic subtypes with classification derived from other omics data is presented in Supplementary Figure.

**Figure 3:**
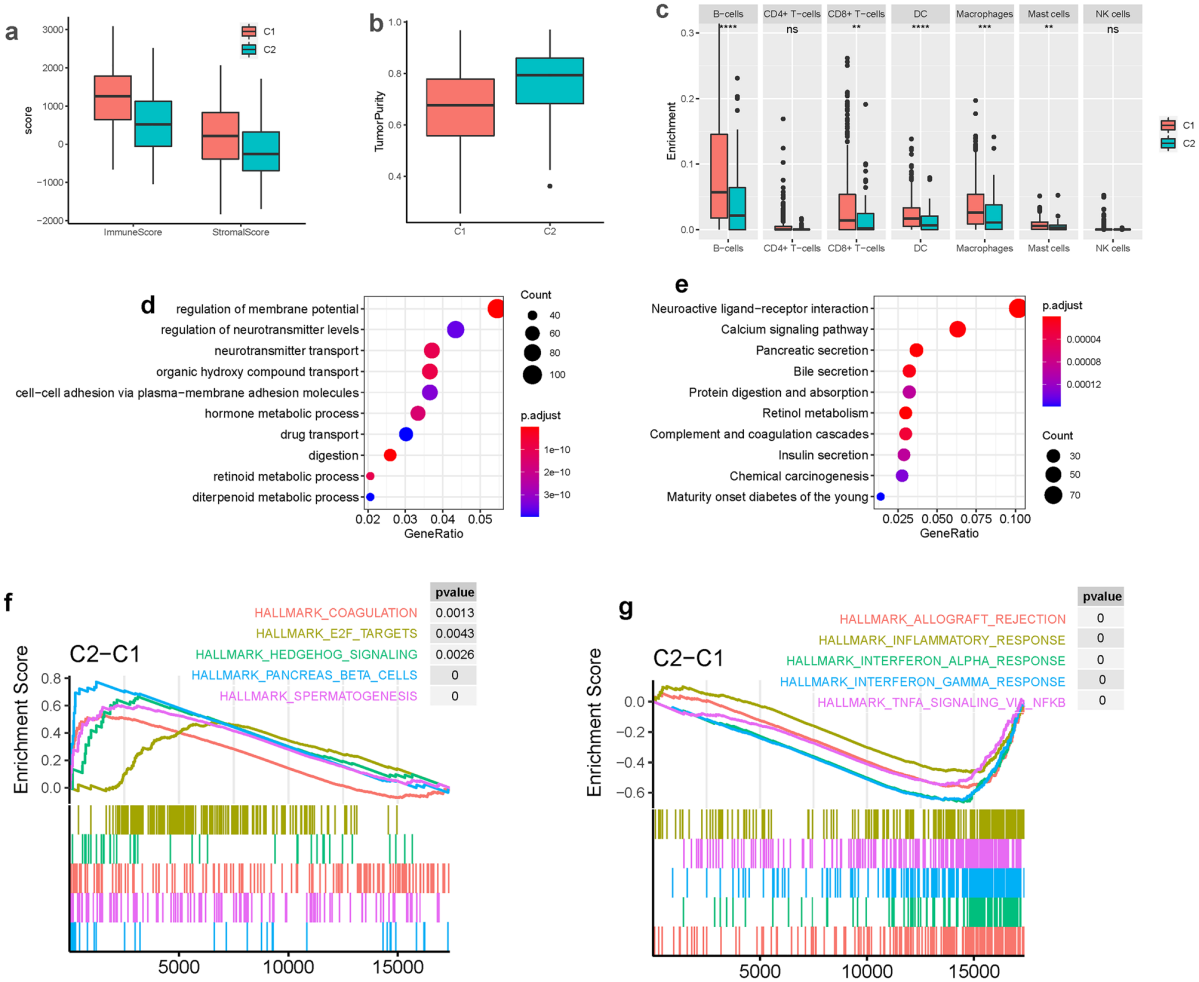
Immune microenvironment characterization and functional enrichment analysis. (**a**, **b**) The immune score, stromal score and tumor purity of different gastric cancer subtypes. (**c**) Boxplot shows the immune microenvironment-related composition of different subtypes. GO (**c**) and KEGG (**d**) enrichment analysis of differentially expressed genes between subtype C1 and C2. GSEA result shows top 5 pathways that are enriched in C1 (**g**) and C2 (**f**) respectively.

Some previously identified subtypes are characterized with specific alterations that may have clinical implication for oncology. For example, MSI subtype is characterized with hypermutated genome. Previous research suggests that mutation burden is significantly associated with immunotherapeutic sensitivity of oncology^22^. Here, subtype N4 and N5 are highly overlapped with MSI subtype, suggesting that they are more likely to respond to immunotherapy which is confirmed in Fig. 5. EBV subtype, presented here by N5, is characterized with high expression of PD1 and PD-L1, suggesting a more sensitive phenotype to immunotherapy that is also confirmed in the following part of immunotherapeutic sensitivity analysis that N5 is significantly associated with responder group to immunotherapy, making it consistent with prediction derived from other EBV subtype. CNV is previously found to be anticorrelated with immune levels^23^, suggesting negative regulatory in oncology. In this study, C2 is presented with CNV high group, suggesting a more inhibited immunity of C2 which is consistent with the observation in Fig. 3a.

### Immune infiltration and functional enrichment analysis of different gastric cancer subtypes

Immune cell infiltration is analyzed to characterize the immune landscape of gastric cancer. We used xCell^24^ to quantify the proportion of 64 immune cells and stromal cells. Tumor purity is evaluated using R package estimate. Comparison between different subtypes shows that subtype C1 has a higher immune score and stromal score than subtype C2 (Fig. 3a). But subtype C1 has lower tumor purity than C2 (Fig. 3b). Furthermore, different immune cell infiltration is analyzed between subtypes C1 and C2. We found that subtype C1 had higher enrichment of B cells, CD8 + T cells, dendritic cells, macrophages, and mast cells than C2 (Fig. 3c). But no obvious difference is observed of CD4 + T cells and NK cells.

To check whether these subtypes are enriched in different biological processes and states. We performed differential expression analysis between C1 and C2 and identified 2117 differentially expressed genes. Gene ontology (GO) enrichment analysis shows that these differentially expressed genes are enriched in various metabolic processes including hormone metabolic process, retinoid metabolic process, and diterpenoid metabolic process (Fig. 3d). In addition to these metabolic processes, digestion, drug transport, and neurotransmitter transport are also represented by these differentially expressed genes. Kyoto Encyclopedia of Genes and Genomes (KEGG) enrichment analysis shows that these differentially expressed genes are enriched in pancreatic secretion, bile secretion, insulin secretion, retinol metabolism, and protein digestion and absorption (Fig. 3e).

In addition, an independent method called gene set enrichment analysis (GSEA)^25^ was used to analyze the functional difference between different subtypes. We collected 50 hallmark gene sets representing well-defined biological states or processes to check whether they are enriched in different pathways. We found that interferon-gamma response, interferon-alpha response, and inflammatory response are enriched in subtype C1. Pancreas beta cells and bile acid metabolism are enriched in subtype C2. The top 5 enriched pathways are presented in Fig. 3f and g.

### Association of different subtypes with genomic alteration

**Figure 4:**
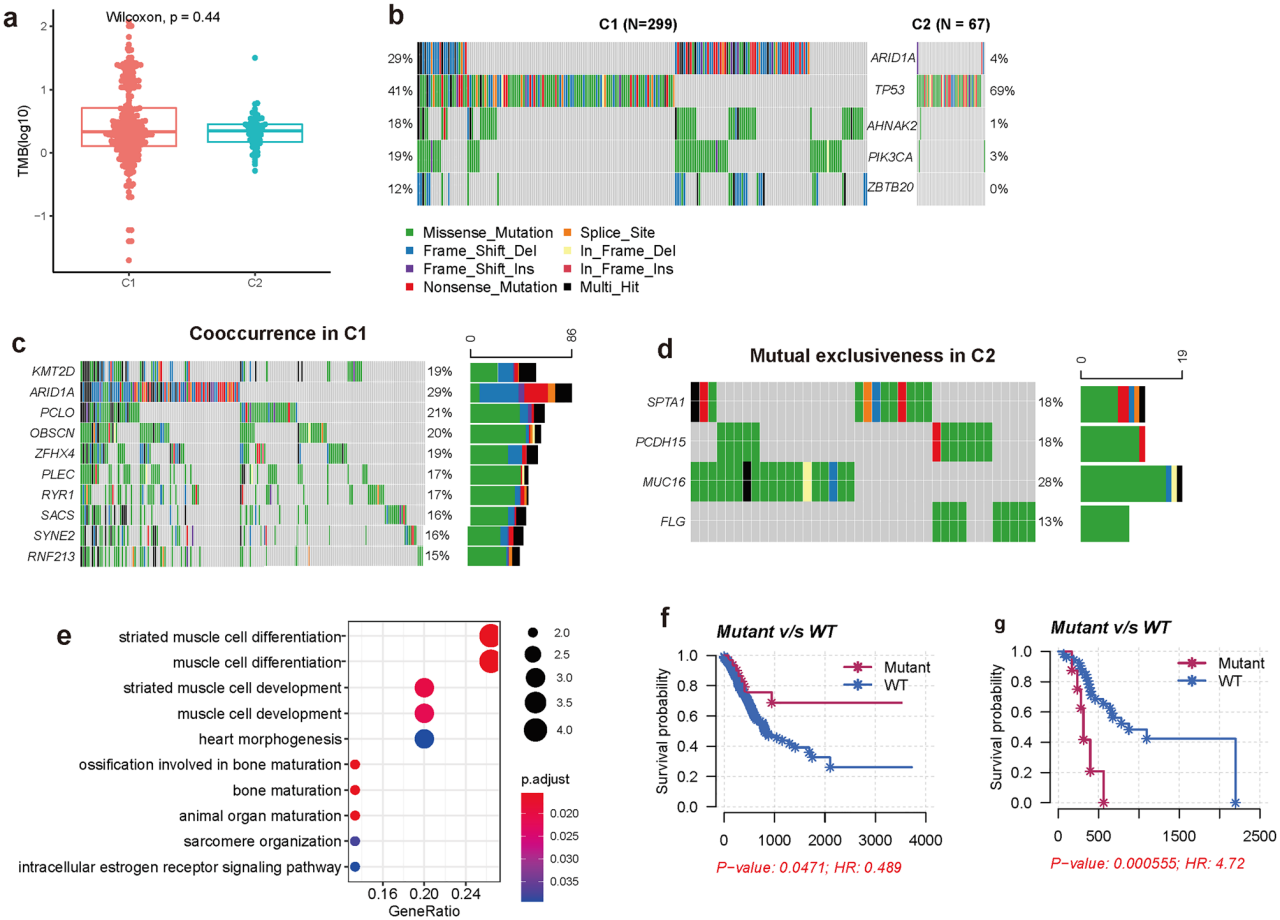
Genomic characterization of different gastric cancer subtypes. (**a**) Boxplot shows tumor mutation burden of different subtypes. (**b**) Differentially mutated genes between subtype C1 and C2. (**c**) Heatmap shows mutational cooccurrence in subtype C1. (**d**) Heatmap shows the mutually exclusive mutations in subtype C2. (**e**) GO enrichment analysis of these co-occurred mutation. Survival analysis between ABCA13 mutant and wild type in subtype C1 (**f**) and C2 (**g**).

Tumor mutation burden (TMB) is found to affect the immunotherapeutic sensitivity of cancer^22^. To check whether there is a difference in mutational frequency between gastric cancer subtypes, We calculated TMB for each sample. Comparative analysis shows that there is no obvious difference in TMB between subtypes C1 and C2 (Fig. 4a). In addition, differentially mutated genes are also explored in our analysis to check whether some genes are preferentially mutated in a specific subtype. Five genes are found to be differentially mutated between subtypes C1 and C2 in our analysis. ARID1A, AHNAK2, PIK3CA, and ZBTB20 are preferentially mutated in C1, and TP53 is preferentially mutated in C2 (Fig. 4b).

Genetic alterations are often grouped into different combinations to drive the development of cancer^26^. These specific genomic events are more or less likely to be co-selected during the initiation and progression of cancer. To study the mutational dependency of different subtypes, we used the SELECT algorithm to identify mutual exclusivity and co-occurrence events of genomic alterations^27^. Genomic analysis of subtype C1 shows that KMT2D mutation is co-occurred with ARID1A, PCLO, OBSCN, AFHX4, PLEC, RYR1, SACS, SYNE2, and RNF213 mutation (Fig. 4c). GO enrichment analysis of these co-occurred mutations shows that they are enriched in muscle cell differentiation, muscle cell development, and heart morphogenesis (Fig. 4e). In addition to KMT2D, TTN mutation is found to be co-occurred with PLEC, SACS, SYNE2, and RNF213 mutation. But no mutual exclusivity of genomic mutation is found in C1. Genomic analysis of subtype C2 shows that SPTA1 mutation is mutually exclusive with PCDH15 mutation and MUC16 mutation is mutually exclusive with FLG mutation (Fig. 4d). But no co-occurred mutation is found in this subtype.

The prognostic value of these significantly mutated genes is also under investigation in our analysis. To check whether these significantly mutated genes contribute to the survival of gastric cancer, we analyzed the top 50 mutated genes for different subtypes. GLI3, LAMA1, and LRRK2 are found to be significantly associated with the survival of gastric cancer in subtype C1. Mutation of these genes tends to be a protective factor. Gastric cancer in subtype C1 carrying the mutant of these genes displayed a favorable survival. Survival analysis of subtype C2 shows that ABCA13, VPS13D, and CUBN affect the survival of gastric cancer. Patients carrying the mutant of these genes displayed a poor survival compared with the wild type. Interestingly, we found that ABCA13 displayed a distinct prognostic value between C1 and C2. Survival analysis of ABCA13 shows that patients in subtype C1 carrying this mutation have better survival than the wild type (Fig. 4f). But we get a distinct result in subtype C2. ABCA13 mutant displayed a poor survival compared with the wild type in C2 (Fig. 4g), suggesting that ABCA13 can be epigenetically regulated by these lncRNAs.

To determine the interactions between ABCA13 and lncRNA based classification. We investigated the association between ABCA13 and these 100 lncRNAs used for classification and found that some lncRNAs tend to be correlated with ABCA13 mutant. For example, gastric cancer patients carrying ABCA13 mutant in C2 group is found to have an upregulated expression of LINC2826, AC25575.2, and AFAP-AS1 than the wild type (Supplementary Fig. 2), but no difference of AC25575.2 and AFAP-AS1 expression is observed in the C1 group, suggesting that ABCA13 is epigenetically regulated by these lncRNAs which collectively affects the survival of gastric cancer.

### Chemotherapeutic and immunotherapeutic sensitivity of different subtypes

**Figure 5:**
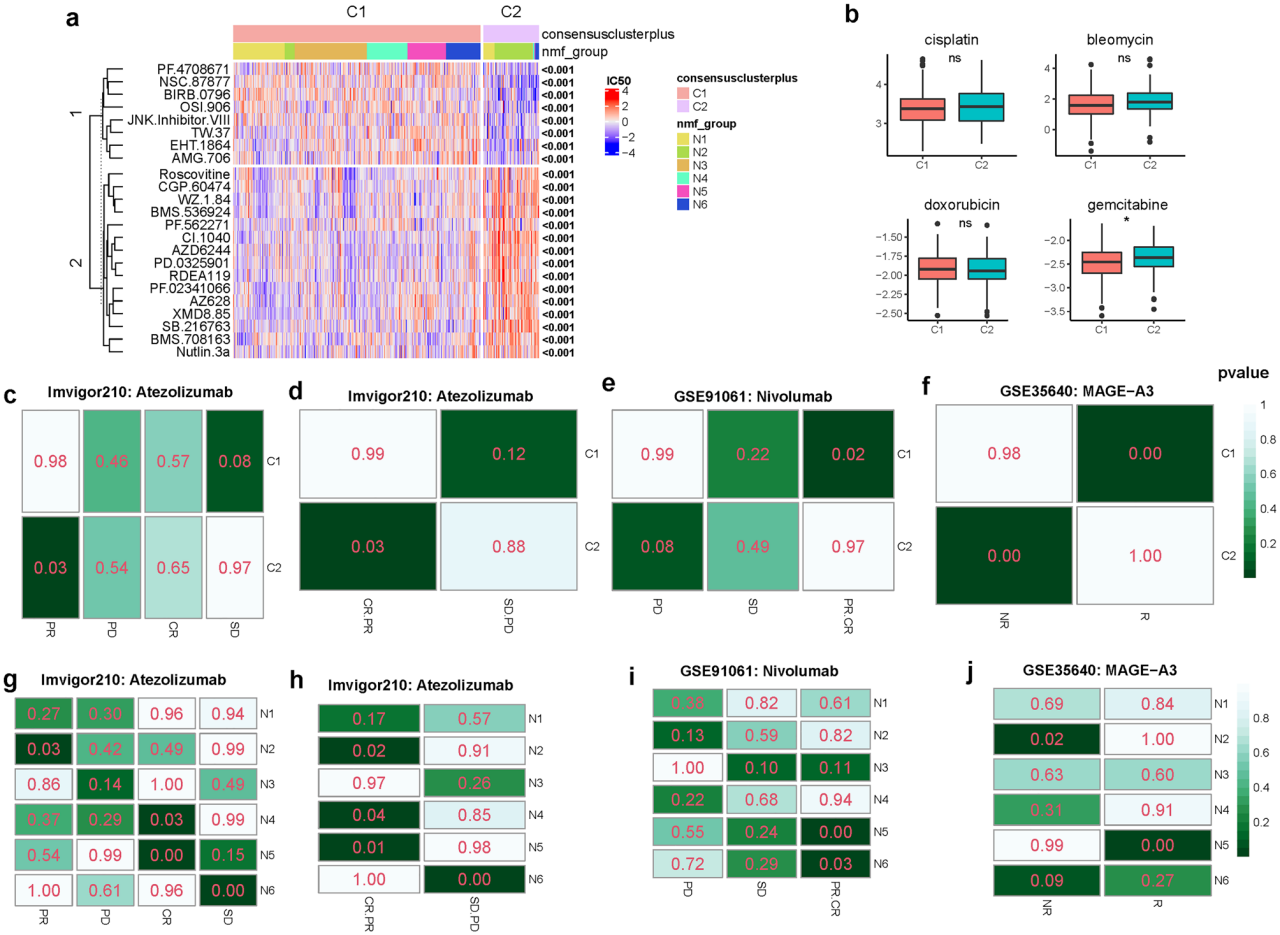
Chemotherapeutic and immunotherapeutic sensitivity of different subtypes. (**a**) Heatmap shows the chemotherapeutic sensitivity (IC50) of gastric cancer subtypes to different chemical drugs. (**b**) Chemotherapeutic sensitivity of four representative chemical drugs between different subtypes. (**c**–**j**) SubMap analysis shows the similarity of gene expression profiles between gastric cancer subtypes and melanoma patients who treated with atezolizumab, nivolumab, and MAGE-A3 specific immunotherapies. CR: complete response. PR: partial response. PD: progressive disease. SD: stable disease. R: responder. NR: non-responder.

To check whether a specific subtype of gastric cancer could benefit from chemotherapies, we choose four representative chemical drugs (bleomycin, doxorubicin, cisplatin, and gemcitabine) to analyze the chemotherapeutic sensitivity of gastric cancer. We constructed a prediction model based on the Genomics of Drug Sensitivity in Cancer (GDSC) dataset and predicted drug sensitivity (IC50) for each sample of these four chemical drugs. We found that subtype C1 was more sensitive to gemcitabine than C2 (Fig. 5b). For the analysis of cisplatin, bleomycin, and doxorubicin, no obvious difference was observed between subtypes. Additionally, we analyzed other 134 chemical drugs between different subtypes. Among these chemical drugs, twenty-three drugs have distinct sensitivity between subtypes C1 and C2. Subtype C2 is more sensitive to PF.4708671, NSC.87877, BIRB.0796, OSI.906, and JNK.inhibitor.VIII (Fig. 5a). Comparing with subtype C2, C1 is more sensitive to Roscovitine, CGP.60474, WZ.1.84, AZ628, and Nutlin.3a.

To investigate whether our gastric cancer subtypes could benefit from immunotherapies, we collected three datasets of melanoma patients who received atezolizumab, nivolumab, and MAGE-A3. Submap^28^ is used to compare gene expression profiles of gastric cancer subtypes and the melanoma patients and calculate the similarity of subclasses across different cohorts and platforms. Submap analysis of Imvigor210 cohort shows that subtype C2 is significantly correlated with partial response group, whereas C1 is associated with stable disease group (Fig. 5c). After combining complete response and partial response into responder group, stable disease and progressive disease into non-responder group, we got a similar result. Subtype C2 is significantly associated with the responder group (Fig. 5d), suggesting that C2 is more sensitive to atezolizumab. Submap analysis of GSE91061 cohort shows that C1 is significantly associated with the responder group, whereas C2 is associated with the progressive disease group (Fig. 5e). Submap analysis of GSE35640 cohort shows that C1 is significantly associated with the responder group but C2 is correlated with the non-responder group (Fig. 5f), suggesting that C1 might respond to MAGE-A3.

In addition, immunotherapeutic sensitivity of NMF subtype is also investigated. Analysis of imvigor210 cohort shows that N2 is significantly associated with the partial response group, N4 and N5 are significantly associated with complete response group, N6 is associated with the stable disease group (Fig. 5g). N2, N4, and N5 are all found to be significantly correlated with the atezolizumab-responder group (Fig. 5h). Analysis of nivolumab shows that N5 and N6 are significantly associated with the responder group (Fig. 5i). For the analysis of MAGE-A3, we find that N5 is significantly associated with responder group, whereas N2 and N6 are associated with non-responder group (Fig. 5j). These findings collectively demonstrate that gastric cancer subtypes are distinct for chemotherapeutic and immunotherapeutic sensitivity.

## Discussion

In this study, we identified metabolism-related lncRNA. Gastric cancer can be subdivided into different subtypes based on these lncRNAs. Subtype N1 is found to have the best survival than other subtypes. Comparative analysis with the previously identified subtypes shows that our gastric cancer subtype is significantly associated with mRNA, miRNA, copy number, and methylation-based subtypes. Functional enrichment analysis shows that they are enriched in distinct biological processes and states. Analysis of chemotherapeutic and immunotherapeutic sensitivity shows that different subtypes are distinct responding to the same chemical drugs.

Gastric cancer is a highly heterogenous disease. Molecular subtype of gastric cancer has been studied in some papers. It can be histologically classified into intestinal and diffuse subtypes based on Lauren classification. Furthermore, gastric cancer can be molecularly divided into EB virus-positive tumors, microsatellite unstable tumors, genomically stable tumors, and tumors with chromosomal instability^21^. These subtypes of gastric cancer are classified based on mRNA expression, miRNA expression, copy number, methylation, and genomic alteration. But lncRNA-based classification of gastric cancer is lacking. In this study, we conducted molecular classification of gastric cancer based on metabolism-related lncRNA. LncRNA-based subtypes of gastric cancer are found to be highly consistent with the subtypes derived from mRNA expression, miRNA expression, copy number, and methylation, suggesting the interconnection of subtypes across different platforms. But the difference between these subtypes suggests that some features are unique in their subtypes. Thus, an integrated molecular classification of gastric cancer combining lncRNA and other omics data is necessary for the future to elucidate the underlying molecular interactions.

Metabolism of gastric cancer has been widely studied in recent years. Low vitamin B is found to be associated with an increased risk of gastric cancer^29^. metabolism-related genes are reported to affect the prognosis of gastric cancer^30^. Furthermore, PHTF2 is found to regulates lipids metabolism in gastric cancer^31^. But most of these studies concentrate on protein-coding genes, the metabolism-related non-coding RNA remains largely unexplored. In this study, we identified metabolism-related lncRNA. A large number of lncRNAs are found to be associated with at least one metabolic pathway, suggesting the universal regulation of lncRNA in metabolic activity. However, only a few lncRNAs are associated with multiple metabolic pathways, suggesting that these lncRNAs are significant regulators for the metabolic pathways. Therefore, these metabolism-related master lncRNA regulators are of great significance to be explored in the future to study the underlying interactions between lncRNA and metabolic activity.

Note that ABCA13 is not a hypermutated gene and there are only 9 samples carrying ABCA13 mutation in C2 group which may leads to an unreliable result statistically due to the limited cases. Thus, further analysis should concentrate on the collection of large-scale nucleotide polymorphism data to validate the prognostic value of ABCA13 in gastric cancer and the underlying regulatory interactions with lncRNA LINC2826, AC25575.2, and AFAP-AS1. Growing number of researches suggest that lncRNA AFAP-AS1 is associated with multiple cancer types by epigenetically regulating various molecules and pathways. For example, AFAP-AS1 is found to promote proliferation and migration of gastric cancer by epigenetically regulating KLF2^32^. Various targets including AUF1, ROCK1 signaling pathway, miR-145 and so on are all reported to be regulated by AFAP-AS1 to promote tumorigenesis. Therefore, the investigation of underlying regulation between AFAP-AS1 and ABCA13 is necessary and urgently needed to be implemented in the future.

Increasing evidence shows that metabolic property is significantly associated with the survival of cancer and affects the therapeutic sensitivity of treatment^14^. In this work, different subtypes of gastric cancer are found to display distinct sensitivity to the same chemical drugs. Identification of the significant lncRNAs that most contribute to therapeutic response is necessary for further analysis. Furthermore, a large-scale investigation of drug sensitivity should be performed for these subtypes. Currently, some signatures are discovered to be significantly associated with the immunotherapeutic sensitivity of gastric cancer. This classification can identify the potential patients precisely who may respond to the immunotherapies when used in combination with other signatures and improve our understanding of lncRNA-mediated metabolic pathway regulation of gastric cancer.

In summary, lncRNA represents a potential regulator of metabolic activity. Deeply understanding the biological function and clinical relevance of these metabolism-related lncRNAs can provide insights into the underlying mechanism of initiation and progression of gastric cancer. These findings can improve our knowledge of metabolic regulation and advance the research of immunotherapy in the clinical management of gastric cancer.

## Method

### Data collection

Multi-omics data including transcriptome data, clinical information, and genomic mutation data of gastric cancer are collected from The Cancer Genome Atlas (TCGA). For the transcriptome data, we downloaded raw count data and Fragments Per Kilobase Million (FPKM) data. FPKM is then transformed to Transcripts Per Kilobase Million (TPM) for the representation of expression. LncRNA and mRNA are annotated using GENCODE tool. The information of previously identified molecular subtypes of gastric cancer is downloaded using R package TCGAbiolinks^33^.

For the identification of metabolism-related lncRNA, we collected metabolic pathways from The Molecular Signatures Database (MSigDB)^34^. A total of 38 metabolic pathways are collected in this work. For analysis of GSEA between different subtypes, a total of 50 hallmark gene sets representing well-defined biological processes and states are also downloaded from MSigDB. For the analysis of immunotherapeutic sensitivity of gastric cancer, advanced melanoma treated with immune checkpoint blockade (GSE91061)^35^, metastatic urothelial cancer treated with an anti-PD-L1 agent (imvigor210)^36^, and metastatic melanoma treated with MAGE-A3 antigen (GSE35640)^37^ are collected in our analysis.

### Identification of metabolism-related lncRNA

To identify metabolism-related lncRNA, metabolic pathways are collected from MSigDB. LncRNA and mRNA expression profiles are prepared to analyze the association between lncRNA and metabolic pathways. Metabolism-related lncRNAs are identified based on a two-step framework. We first identified differentially expressed lncRNAs between gastric cancer and the adjacent normal samples based on pvalue and absolute fold change. A lncRNA is identified as differentially expressed if BH-adjusted *P* value < 0.05 and absolute fold change > 2. Differential expression analysis of lncRNA is conducted using R package edgeR^38^. The association between these differentially expressed lncRNAs and metabolic pathways is then evaluated using a previously proposed tool ImmLnc^11^. It calculates an association score for each pair of lncRNA and metabolic pathway. After setting a threshold for the association, the pairs surpassing the threshold are identified as significantly associated.

### Identification of molecular subtypes based on metabolism-related lncRNA

Metabolism-related lncRNAs are used to identify molecular subtypes of gastric cancer. We choose the top 100 variable lncRNAs based on standard deviation as features (Supplementary Table 2). R package ConsensusClusterPlus is used to classify gastric cancer into different subtypes. The optimal number of subtypes is determined based on the cumulative distribution function curve and consensus matrix between 2 and 8 subtypes. To check whether the molecular classification of gastric cancer is reliable or not, an independent approach NMF is used to classify gastric cancer. Similarly, cophenetic scores, silhouette width, and residual sum of squares are evaluated to identify the optimal number of clusters. NMF clustering is conducted using R package NMF.

### Functional enrichment analysis

To analyze the different biological processes between subtypes, we identified differentially expressed genes between subtypes C1 and C2. Raw count data of mRNA is downloaded as input for differential expression analysis. A gene is identified as differentially expressed if BH-adjusted *P* value < 0.05 and absolute fold change > 2. KEGG and GO enrichment analysis are used to analyze the functional enrichment of these differentially expressed genes. Enrichment analysis is conducted using R package clusterProfiler. Additionally, GSEA is also used to analyze the functional enrichment between subtypes. We rank the genes based on the fold change. These genes are inputted into GSEA. A total of 50 hallmark gene sets representing well-defined biological processes and states are also inputted into GSEA to analyze whether these genes are enriched in these biological processes. GSEA is conducted using R package enrichplot.

### Mutational analysis of gastric cancer subtypes

For mutation analysis, differentially mutated genes are identified by comparing the mutational frequency between different subtypes. TMB is measured as non-silent mutations per Mb. Identification of survival-related mutation is based on the cox regression model. All of these analyses are conducted using R package maftools^39^. Cooccurrence and mutual exclusivity events of mutation are identified using a previously proposed approach SELECT.

### Analysis of chemotherapeutic and immunotherapeutic sensitivity

For the analysis of chemotherapeutic sensitivity of gastric cancer, we constructed a prediction model based on the GDSC cell line dataset using ridge regression. The sensitivity (IC50) for each sample is evaluated using this prediction model for a specific chemical drug. We calculated the sensitivity of gastric cancer for a total of 138 chemical drugs. These analyses are conducted using R package pRRophetic^40^.

For the analysis of immunotherapeutic sensitivity of gastric cancer, submap is used to compare gene expression profiles between different subtypes. We compared our lncRNA subtypes with melanoma patients who received immunotherapies to check whether a specific subtype is significantly associated with the responder group.

## Supplementary Information

Supplementary Figure S1.

Supplementary Figure S2.

Supplementary Tables.

## Data Availability

All data used in this study are collected from online database.
