# Supplementary figures and images for: Molecular characterization of metabolic subtypes of gastric cancer based on metabolism-related lncRNA
Source: Sci Rep. 2021 Nov 2;11:21491. doi: 10.1038/s41598-021-00410-7 (PMC8563741; doi:10.1038/s41598-021-00410-7)

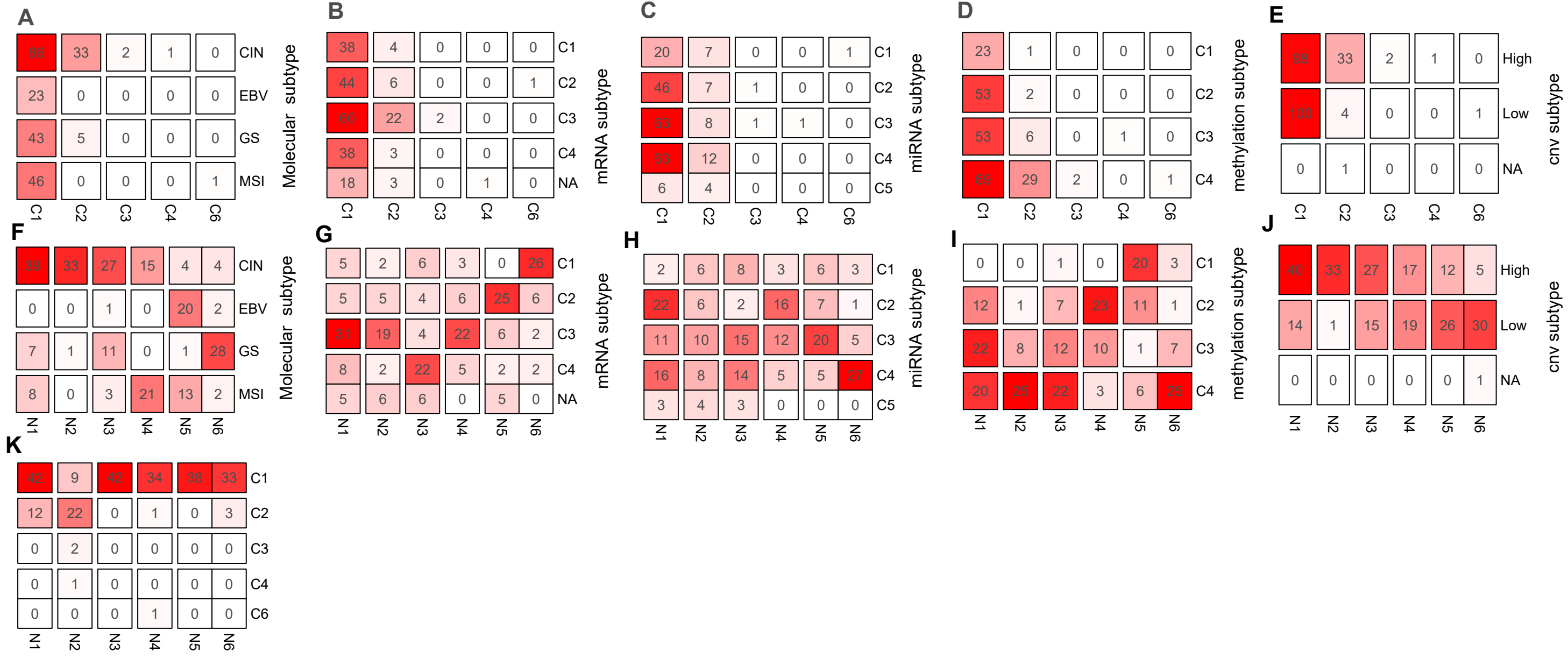

Supplement: Supplementary file 1 — Supplementary Figure S1. [file 41598_2021_410_MOESM1_ESM.pdf]

AFAP-AS1

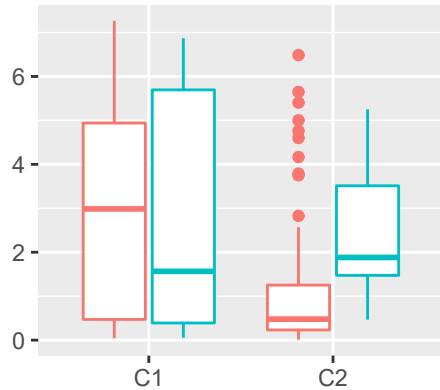

AC025575.2

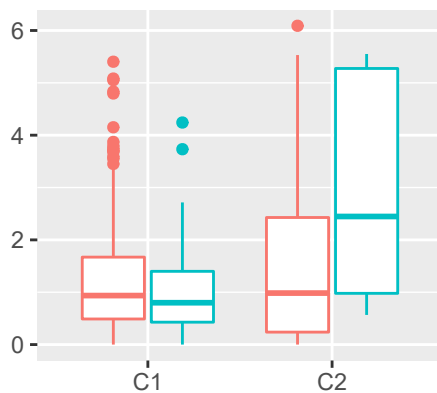

LINC02826

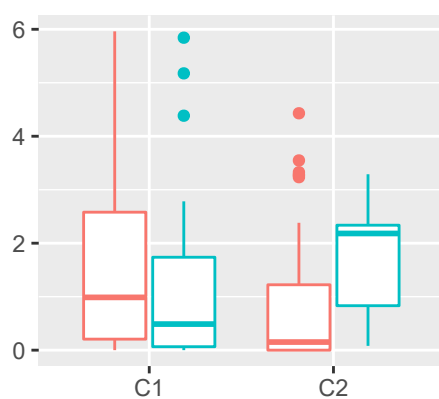

Wild type  
Mutant

Supplement: Supplementary file 2 — Supplementary Figure S2. [file 41598_2021_410_MOESM2_ESM.pdf]
